# Differential effects of the *APOE* e4 allele on different domains of cognitive ability across the life-course

**DOI:** 10.1038/ejhg.2015.210

**Published:** 2015-09-23

**Authors:** Riccardo E Marioni, Archie Campbell, Generation Scotland, Caroline Hayward, David J Porteous, Ian J Deary

**Affiliations:** 1 Centre for Cognitive Ageing and Cognitive Epidemiology, University of Edinburgh, Edinburgh, UK; 2 Medical Genetics Section, Centre for Genomic and Experimental Medicine, Institute of Genetics and Molecular Medicine, University of Edinburgh, Edinburgh, UK; 3 Queensland Brain Institute, The University of Queensland, Brisbane, QLD, Australia; 4 A collaboration between the University Medical Schools and National Health Service in Aberdeen, Dundee, Edinburgh and Glasgow, UK; 5 Medical Research Council Human Genetics Unit, Institute of Genetics and Molecular Medicine, University of Edinburgh, Edinburgh, UK; 6 Department of Psychology, University of Edinburgh, Edinburgh, UK

## Abstract

The association between *APOE* genotype and cognitive function suggests a positive role for the e2 allele and a negative role for the e4 allele. Both alleles have relatively low frequencies in the general population; hence, meta-analyses have been based on many small, heterogeneous studies. Here, we report the *APOE*-cognition associations in the largest single analysis to date. *APOE* status and cognitive ability were measured in 18 337 participants from the Generation Scotland study between 2006 and 2011. The age range was 18–94 years with a mean of 47 (SD 15). Four cognitive domains were assessed: verbal declarative memory (paragraph recall), processing speed (digit symbol substitution), verbal fluency (phonemic verbal fluency), and vocabulary (Mill Hill synonyms). Linear regression was used to assess the associations between *APOE* genetic status and cognition. Possession of the e4 allele was associated with lower scores on the measures of memory and processing speed in subjects aged >60. Across all age ranges, the e4 allele was linked to better verbal fluency scores. In younger subjects (≤60 years) the e4 allele was linked to higher vocabulary scores. There were no associations between the e2 allele and cognitive ability. As seen in previous meta-analyses, the *APOE* e4 allele is linked to poorer cognitive performance in the domains of memory and processing speed. By contrast, positive associations were seen between the e4 allele and measures of verbal fluency and vocabulary. All associations were relatively small and, in many cases, nominally significant despite the very large sample size.

## Introduction

The association between Alzheimer's disease (AD) and the *APOE* e4 allele is well established. It is estimated that individuals who carry two copies of the e4 allele have more than 10 times the risk of developing the late-onset version of the disease.^1^ Although AD typically manifests in older individuals, it is also of interest to investigate the association between *APOE* and cognitive ability across the life-course. Studies have indicated a link between the e4 allele and both lower ability^2^ and increased cognitive decline,^3, 4^ although this is not always the case.^5^ Conversely, the e2 allele is thought to be protective for AD,^6^ and has been associated with longevity and survival in the oldest old.^7^ However, a recent large study (total *n*=2013) of *APOE* genotype and cognitive decline found no association between either e2 or e4 status and cognitive change in five different tests, even when split by age group.^8^ However, the low frequency of both the e2 and e4 alleles (6% and 15%, respectively in the general population^9^) means that these results are based on relatively small numbers.

To provide a more definitive summary of the relationship between *APOE* and cognitive ability requires either a meta-analytic approach^2, 4^ or the analysis of extremely large populations. A previous meta-analysis containing over 40 000 people showed a negative cross-sectional association between the e4 allele and measures of episodic memory, executive function, perceptual speed, and global cognitive ability.^2^ The analysis data came from 77 studies where the largest sample size was 7895, with only eight cohorts having a sample size >1000. However, 34 cohorts had fewer than 100 participants. In addition to the heterogeneity of these populations, there were also differences with the phenotypic measurement as different tests were used to assess the domains under investigation, not all of which were present in all studies. Moreover, there was reduced power to investigate *APOE* zygosity effects and effects of the e2 allele on memory and global ability, with information only available from a maximum of six studies. Age moderation was seen for the domains of episodic memory and global ability, where the negative effect of the e4 allele was more pronounced in older individuals.

Here we overcome the issues of phenotypic and population heterogeneity, and small sample sizes with respect to zygosity and e2 analyses by examining the association between *APOE* and cognitive function in an ethnically and culturally homogeneous sample of 18 337 subjects, aged between 18 and 94 years from the Generation Scotland: Scottish Family Health Study.

## Materials and methods

Data came from Generation Scotland: the Scottish Family Health Study (GS:SFHS), which is a family-based cohort study sampled from the general population in Scotland (www.generationscotland.org/). Details of the study design have been reported previously.^10, 11^ In brief, over 24 000 subjects were recruited into the study between 2006 and 2011. The initial sample of probands (*n*=7953) were registered with general medical practitioners from five regions of Scotland, and aged between 35 and 65 years. Ascertainment was unbiased, ie, there was no recruitment drive to obtain individuals with any particular disorder. Probands' relatives were recruited to yield the full GS:SFHS sample (age range 18–99). There were up to three generations per family with 5628 families and 1395 singleton participants. In the current analysis we only considered individuals who had both *APOE* and cognitive data on at least one test available (*n*=18 337).

### Genotyping sample

*APOE* haplotype status depends on the genotypes of two single-nucleotide polymorphisms, rs429358 (hg19 chr19:g.45411941T>C) and rs7412 (hg19 chr19:g.45412079C>T). These two single-nucleotide polymorphisms can form three possible haplotypes: e2=hg19 chr19:g.[45411941T=45412079C>T] (rs429358 T and rs7412 T), e3=hg19 chr19:g.[45411941T=45412079C=] (rs429358 T and rs7412 C) and e4=hg19 chr19:g.[45411941T>C=45412079C=] (rs429358 C and rs7412 C).^12^ Array genotyping of these single-nucleotide polymorphisms is technically difficult and, as a result, they are not available on the majority of commercial arrays. single-nucleotide polymorphism genotypes were thus obtained using Taqman technology at the Wellcome Trust Clinical Research Facility Genetics Core, Edinburgh. Blood samples from GS:SFHS participants were collected, processed and stored using standard operating procedures and managed through a laboratory information management system at the Wellcome Trust Clinical Research Facility Genetics Core, Edinburgh.^13^

### Ethics statement

All components of GS:SFHS received ethical approval from the NHS Tayside Committee on Medical Research Ethics (REC Reference Number: 05/S1401/89). GS:SFHS has also been granted Research Tissue Bank status by the Tayside Committee on Medical Research Ethics (REC Reference Number: 10/S1402/20), providing generic ethical approval for a wide range of uses within medical research.

### Cognition

Four cognitive ability domains were assessed in GS:SFHS – processing speed (Wechsler Digit Symbol Substitution Test – DST^14^), verbal declarative memory (Wechsler Logical Memory Test – LM; sum of immediate and delayed recall of one paragraph^15^), verbal fluency (the phonemic Verbal Fluency Test – VFT; using the letters C, F, and L, each for 1 min^16^), and vocabulary (the Mill Hill Vocabulary Scale – MHVS; junior and senior synonyms combined^17^). A general cognitive ability factor, g, was obtained via a principal components analysis of the four test scores. The first unrotated principal, which explained 42% of the variance, was extracted. All four tests loaded strongly on g (range 0.58–0.71).

### Covariates

In addition to age and sex in the baseline models of *APOE* e4 status and cognitive ability, sensitivity analyses were conducted that adjusted for potential confounding and mediating variables. These included education, social class, and self-reported heart disease, history of stroke, high blood pressure, diabetes, AD, and depression. Education was recorded using an ordinal scale that ranged from 0 to 10, where each value represented a 'bin' for different numbers of years of full-time education (0, 1–4, 5–9, 10–11, 12–13, 14–15, 16–17, 18–19, 20–21, 22–23, ≥24 years). Social class was measured using the Scottish Index of Multiple Deprivation 2009 (http://www.scotland.gov.uk/topics/statistics/simd/). The Scottish Index of Multiple Deprivation ranks small areas of Scotland using information on seven domains: income, employment, health, education, geographic access, crime, and housing. The range of Scottish Index of Multiple Deprivation ranks is from 1 (most deprived) to 6505 (least deprived).

### Statistical analyses

Age-, and sex-adjusted linear mixed effects models of *APOE* against the cognitive measures were performed for three different parameterisations of *APOE*: e4 present *versus* absent; e4 dose – 0, 1, or 2 alleles; and *APOE* haplotype – e2e2, e2e3, e2e4, e3e4, e4e4, with e3e3 as the reference level. Pedigree information was used to define relatedness. Genetic relationships between family members can be derived theoretically. For example, the genetic correlation between parent–offspring pairs is 0.5, for grandparent–grandchildren it is 0.25, for first cousins it is 0.12. A kinship matrix including this information conditions the random additive genetic effect in the model.

To investigate age-stratified differences the cohort was split at 60 years to see if there were differential effects of *APOE* in later life compared to mid-life. The threshold of 60 years was chosen as an arbitrary cut-point for older age. A continuous age – *APOE* interaction was also examined. Sensitivity analyses adjusting for additional covariates were also performed. All models were run in the statistical software package 'R', utilising the 'asreml' library.^18, 19^ Wald tests were used to calculate *P*-values.

### Data availability

GS data are available at the European Genome-phenome Archive (https://www.ebi.ac.uk/ega/home) under accession number EGAS00001001235.

## Results

Table 1 shows the characteristics of the GS:SFHS cohort. Of the 18 337 individuals (41% male) included in the analyses, the mean age was 47 (SD 15) years. The majority of the population carried two copies of the e3 allele (*n*=10 926, 60%) with 469 (2.6%) individuals having the e4e4 haplotype and 111 (0.6%) having the e2e2 haplotype. The proportions of e2e3, e2e4, and e3e4 haplotypes were 12.0%, 2.2%, and 23.1% (*n*=2194, 411, 4226), respectively. There was very little difference in the distribution of *APOE* alleles for those older than 60 years compared with those 60 or younger (Supplementary Table 1). The distributions of cognitive scores by *APOE* status are presented in Supplementary Table 2.

**Figure 1:**
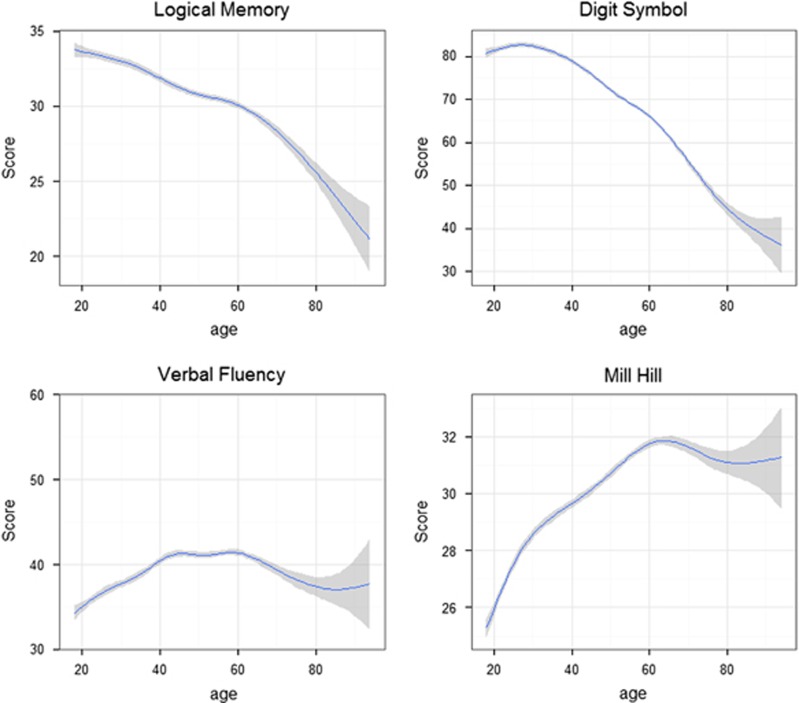
Distribution of the four cognitive tests by age.

Of the self-reported health variables, high blood pressure was the most prevalent (13%), followed by depression (10%). The prevalences of the other conditions were below 4% with only 23 (0.1%) individuals reporting AD. The cross-sectional distributions of the four cognitive tests by age are presented in Figure 1. There is marked 'decline' in scores on both the digit symbol and logical memory tasks with age. The vocabulary measure 'increases' in early adulthood before remaining relatively stable over the remainder of the life-course. There is an inverted U-shaped pattern for verbal fluency scores with lower scores in young and late adulthood.

The output from the regression models are shown in Table 2. Having two e4 alleles was associated with a one-point decrease (standardised beta (*β*)=−0.12, *P*=0.007) in memory score relative to individuals who had the e3e3 haplotype. Having either an e3e4 or e4e4 haplotype was associated with around a one-point increase in the measure of verbal fluency (*β*=0.064, *P*=0.001, and *β*=0.092, *P* =0.050, respectively). The e3e4 haplotype was also associated with a very small increase in the vocabulary test score (*β*=0.044, *P*=0.015). There was no evidence of any effect – protective or otherwise – of having one or two e2 alleles (Supplementary Table 3). There were no strong associations between g and *APOE* status other than a slightly increased cognitive score for e4 carriers, which was more notable in the younger (≤60 years) sub-population (Supplementary Table 4).

Table 3 presents analyses that stratified by age (≤60 years and >60). In the older subset of the cohort there was a pronounced effect of the e4 allele on memory and processing speed with e4e4 carriers scoring on average 2.6 and 4.2 points less, respectively, than e3e3 individuals on these tests (*β*=−0.32, *P*=0.003 and *β*=−0.27, *P*=0.006). The e4 additive effects were associated with lower scores on memory (0.8 points per allele, *β*=−0.095, *P*=0.003) and processing speed (1.3 points per allele, *β*=−0.087, *P*=0.004); the presence (*versus* absence) of the e4 allele was associated with similar effect sizes. There were no significant effects of e4 allele possession on these test scores in the younger subset. Only those aged 60 years or younger retained the association between the e3e4 (*versus* e3e3) haplotype and improved verbal fluency test scores and vocabulary. The positive association between the e4e4 haplotype and verbal fluency persisted in the older age group, with an increased mean test score of 2.7 points over those with the e3e3 haplotype (*β*=0.232, *P*=0.034). The additive effect of the e4 allele on higher verbal fluency scores was seen in both sub-groups; the effect sizes for the presence *versus* absence of the e4 allele were similar in both sub-groups but were only significant in the younger participants. Significant additive and presence *versus* absence effects of the e4 allele were only seen in the younger sub-group for the vocabulary measure. The models that investigated a continuous interaction between age and *APOE* status yielded significant findings for verbal fluency only (Supplementary Table 5). Test scores increased by 0.03 SDs for e4 carriers per additional year (*P*=0.034).

The sensitivity analyses that included full covariate adjustment made little difference to the effect size estimates (Supplementary Tables 6 and 7). There was no association between the *APOE* e2 allele and any of the cognitive test scores in an age- and sex-adjusted analysis (Supplementary Table 2).

## Discussion

This study is the largest single analysis of *APOE* genotype and cognition to date. For those over the age of 60, possession of the e4 allele was associated with lower memory and processing speed scores. Conversely, across all age ranges, the e4 allele was associated with improved performance on the test of verbal fluency. There were also modest associations between positive e4 carrier status and higher vocabulary scores in the younger participants (<60). All of these associations were retained after controlling for cardiovascular, mental health, and demographic variables. There were no associations between the *APOE* e2 allele and cognitive scores on any of the tests.

The main strengths of this study are its large sample size that enabled us to detect relatively small effects, the cognitive battery testing a number of domains, and wide age range, which allowed us to test for moderation effects. Limitations include the cross-sectional nature of the cognitive data, which restricted us to an analysis of *APOE* and a range of cognitive abilities, but not cognitive decline.^3, 4^ Other limitations include the arbitrary cut-point of 60 years for the age stratification, which may obscure selectivity, eg, distribution of alleles. However, despite the large sample size, there were relatively low numbers of e2 and e4 carriers upon stratification. The paucity of rare allele carriers is also apparent from the results tables where sub-group *P*-values are relatively modest.

Interpretation of the results is relatively straightforward for the domains of memory and processing speed, where the e4 allele was associated with lower test scores, which is in the same direction as those previously reported.^2^ The positive association between the e4 allele and verbal fluency scores fits less well with the existing literature.^2^ However, the distribution of the verbal fluency scores in Figure 1 is quite different to those for digit symbol and logical memory. Indeed, it resembles more closely the 'crystallised' vocabulary-based measure, which may explain the divergent findings from the 'fluid' tests, whose distributions mirror those previously described.^20^ The small positive associations between the e4 allele and vocabulary in the younger members of the cohort also support the differential effects of *APOE* on crystallised-type and fluid-type measures of intelligence.

The magnitude of the effect sizes observed in the current study are in line with those reported from a previous meta-analysis.^2^ The maximum standardised effect size in the current study for the e4 *versus* no e4 analysis, which is analogous to that reported by Wisdom *et al.*, was –0.09 for the association with memory in the older (>60 years) sub-group for the logical memory test. The other effect sizes in the older group were 0.07 (verbal fluency), –0.08 (digit symbol), and –0.03 (Mill Hill). In the younger group the effect sizes were 0.05 for both verbal fluency and Mill Hill, and –0.006 and –0.004 for logical memory and digit symbol, respectively. The lack of any associations between the *APOE* e2 allele and cognition is in accordance with the meta-analysis findings of Wisdom, although the number of e3 carriers in GS:SFHS was an order of magnitude greater than the equivalent number in the meta-analysis.

In conclusion, we found negative associations between the *APOE* e4 allele and memory and processing speed in later life. The magnitude of the effects is consistent with those previously reported. The e4 allele was also linked to improved verbal fluency, which goes against our *a priori* hypothesis and some existing evidence, although this can potentially be explained by the distribution of the scores, which resemble more closely the shape of what one would expect from a 'crystallised'-type measure. There was no relationship between cognitive performance and the *APOE* e2 allele.

## Figures and Tables

**Table 1:** Characteristics of the Generation Scotland: Scottish Family Health Study cohort (*n*=18 337)

	*Mean*	*SD*
Age (years)	47.2	15.0
Education^a^	4.7	1.6
		
*Cognitive (number of correct items)*		
DST	72.3	171
VFT	39.8	11.7
MHVS	30.1	4.7
LM	31.1	7.9

	*Median*	*IQR*
SIMD	4342	2370–3910

	n	*%*
*APOE*		
e2e2	111	0.6
e2e3	2194	12.0
e2e4	411	2.2
e3e3	10 926	59.6
e3e4	4226	23.1
e4e4	469	2.6
Sex (male)	7506	40.9
Heart disease (yes)	638	3.5
Stroke (yes)	232	1.3
High blood pressure (yes)	2421	13.2
Diabetes (yes)	570	3.1
Alzheimer's disease (yes)	23	0.1
Depression (yes)	1752	9.6

Abbreviations: DST, Digit Symbol Test; LM, logical memory; MHVS, Mill Hill Vocabulary Scale; VFT, Verbal Fluency Test.

a Education was measured as an ordinal variable (*n*=17 687). 0: 0 years (*n*=7), 1: 1–4 years (*n*=51), 2: 5–9 years (*n*=510), 3: 10–11 years (*n*=4781), 4: 12–13 years (*n*=3858), 5: 14–15 years (*n*=2594), 6: 16–17 years (*n*=3525), 7: 18–19 years (*n*=1727), 8: 20–21 years (*n*=445), 9: 22–23 years (*n*=127), 10: ≥24 years (*n*=62).

**Table 2:** Associations between *APOE* and cognitive ability adjusted for age and sex

	*Logical memory*			*Verbal fluency test*			*Digit symbol test*			*Mill Hill vocabulary scale*		
	*Beta*	*SE*	P	*Beta*	*SE*	P	*Beta*	*SE*	P	*Beta*	*SE*	P
e4 (additive)	−0.027	0.015	0.067	**0.055**	**0.015**	**<0.001**	−0.014	0.013	0.288	**0.036**	**0.014**	**0.011**
e4 (yes *vs* no)	−0.020	0.017	0.238	**0.062**	**0.017**	**<0.001**	−0.011	0.015	0.457	**0.044**	**0.016**	**0.006**
e2e2	−0.028	0.093	0.914	−0.024	0.094	0.745	−0.057	0.082	0.428	−0.029	0.088	0.633
e2e3	−0.039	0.023	0.181	−0.018	0.024	0.142	0.035	0.021	0.066	0.002	0.022	0.583
e2e4	−0.044	0.049	0.495	−0.025	0.050	0.294	−0.020	0.043	0.689	0.056	0.046	0.399
e3e4	−0.015	0.018	0.664	**0.064**	**0.018**	**0.001**	−0.0002	0.016	0.854	**0.044**	**0.017**	**0.015**
e3e3	—	—	—	—	—	—	—	—	—	—	—	—
e4e4	**−0.123**	**0.046**	**0.007**	**0.092**	**0.047**	**0.050**	−0.048	0.041	0.233	0.036	0.044	0.405

Abbreviations: Beta, standardised beta; P, incremental *P*-value; SE, standard error. *P*-values <0.05 are highlighted in bold.

**Table 3:** Age-stratified (>60 and ≤60) associations between *APOE* and cognitive ability adjusted for age and sex

	*Logical memory*			*Verbal fluency test*			*Digit symbol test*			*Mill Hill vocabulary scale*		
	*Beta*	*SE*	P	*Beta*	*SE*	P	*Beta*	*SE*	P	*Beta*	*SE*	P
*Age >60 years*												
e4 (additive)	**−0.095**	**0.033**	**0.003**	**0.075**	**0.033**	**0.023**	**−0.087**	**0.030**	**0.004**	**−**0.026	0.033	0.423
e4 (yes *vs* no)	**−0.090**	**0.037**	**0.016**	0.070	0.038	0.064	**−0.082**	**0.034**	**0.017**	**−**0.025	0.038	0.504
e2e2	0.075	0.225	0.614	**−**0.219	0.227	0.317	**−**0.205	0.206	0.352	0.020	0.224	0.928
e2e3	**−**0.079	0.051	0.326	**−**0.049	0.051	0.181	0.032	0.047	0.220	0.026	0.051	0.515
e2e4	**−**0.087	0.112	0.606	0.055	0.113	0.738	**−**0.079	0.103	0.583	**−**0.016	0.113	0.930
e3e4	**−**0.080	0.041	0.096	0.042	0.041	0.425	**−**0.057	0.038	0.219	**−**0.015	0.041	0.770
e3e3	—	—	—	—	—	—	—	—	—	—	—	—
e4e4	**−0.321**	**0.108**	**0.003**	**0.232**	**0.110**	**0.034**	**−0.272**	**0.100**	**0.006**	**−**0.076	0.109	0.484
												
*Age* ≤*60 years*												
e4 (additive)	**−**0.013	0.016	0.425	**0.044**	**0.016**	**0.008**	**−**0.007	0.015	0.638	**0.042**	**0.015**	**0.007**
e4 (yes *vs* no)	**−**0.006	0.019	0.748	**0.054**	**0.019**	**0.004**	**−**0.004	0.017	0.806	**0.050**	**0.018**	**0.004**
e2e2	**−**0.031	0.104	0.830	**−**0.020	0.104	0.769	**−**0.046	0.098	0.560	**−**0.048	0.097	0.534
e2e3	**−**0.023	0.026	0.443	**−**0.003	0.027	0.477	0.045	0.025	0.066	**−**0.006	0.025	0.350
e2e4	**−**0.033	0.055	0.597	**−**0.031	0.055	0.316	**−**0.031	0.051	0.527	0.070	0.051	0.305
e3e4	**−**0.0005	0.020	0.816	**0.063**	**0.020**	**0.002**	0.009	0.019	0.551	**0.047**	**0.019**	**0.021**
e3e3	—	—	—	—	—	—	—	—	—	—	—	—
e4e4	**−**0.085	0.051	0.098	0.039	0.051	0.443	**−**0.031	0.048	0.522	0.050	0.048	0.296

Abbreviations: Beta, standardised beta; P, incremental *P*-value; SE, standard error. *P*-values <0.05 are highlighted in bold.
