# Supplementary material for: Differential effects of the APOE e4 allele on different domains of cognitive ability across the life-course
Source: Eur J Hum Genet. 2015 Sep 23;24(6):919–23. doi: 10.1038/ejhg.2015.210 (PMC4705436; doi:10.1038/ejhg.2015.210)
Supplement: Supplementary Tables [file ejhg2015210x1.docx]

**Supplementary Tables**

Supplementary Table 1: *APOE* distribution stratified by age

| ***APOE*** | **n (**≤**60)** | **%** | **n (>60)** | **%** |
| --- | --- | --- | --- | --- |
| e2e2 | 92 | 0.6 | 19 | 0.5 |
| e2e3 | 1 731 | 11.8 | 463 | 12.5 |
| e2e4 | 329 | 2.3 | 82 | 2.2 |
| e3e3 | 8 665 | 59.3 | 2 261 | 60.9 |
| e3e4 | 3 425 | 23.4 | 801 | 21.6 |
| e4e4 | 382 | 2.6 | 87 | 2.3 |

Supplementary Table 2: Distribution of cognitive scores by *APOE* genotype

|  |  |  |  |  |  |  |  |  |
| --- | --- | --- | --- | --- | --- | --- | --- | --- |
|  | Logical Memory | | Verbal Fluency | | Digit Symbol | | Mill Hill Vocabulary | |
| *APOE* status | Mean | SD | Mean | SD | Mean | SD | Mean | SD |
| e4 non-carrier | 31.1 | 7.9 | 39.6 | 11.7 | 72.3 | 17.1 | 30.1 | 4.7 |
| e4 carrier | 31.0 | 7.9 | 40.3 | 11.7 | 72.4 | 17.0 | 30.2 | 4.7 |
|  |  |  |  |  |  |  |  |  |
| 0 e4 alleles | 31.1 | 7.9 | 39.6 | 11.7 | 72.3 | 17.1 | 30.1 | 4.7 |
| 1 e4 allele | 31.1 | 7.9 | 40.3 | 11.7 | 72.5 | 17.0 | 30.2 | 4.8 |
| 2 e4 alleles | 30.3 | 8.0 | 40.4 | 11.8 | 71.4 | 16.9 | 30.2 | 4.6 |
|  |  |  |  |  |  |  |  |  |
| e2e2 | 31.7 | 7.5 | 39.2 | 11.9 | 73.3 | 18.5 | 29.8 | 4.9 |
| e2e3 | 30.8 | 7.8 | 39.5 | 11.4 | 72.9 | 16.9 | 30.1 | 4.7 |
| e2e4 | 30.9 | 8.3 | 39.4 | 12.0 | 71.8 | 16.8 | 30.4 | 5.0 |
| e3e3 | 31.1 | 8.0 | 39.7 | 11.8 | 72.2 | 17.1 | 30.1 | 4.7 |
| e3e4 | 31.1 | 7.9 | 40.4 | 11.7 | 72.6 | 17.1 | 30.2 | 4.7 |
| e4e4 | 30.3 | 8.0 | 40.4 | 11.8 | 71.4 | 16.9 | 30.2 | 4.6 |

SD: standard deviation

Supplementary Table 3: Associations between *APOE* e2 and cognitive ability adjusted for age, and sex.

|  | **Logical Memory** | | | **Verbal Fluency Test** | | | **Digit Symbol Test** | | | **Mill Hill Vocabulary Scale** | | |
| --- | --- | --- | --- | --- | --- | --- | --- | --- | --- | --- | --- | --- |
|  | Beta | SE | P | Beta | SE | P | Beta | SE | P | Beta | SE | P |
| All ages |  |  |  |  |  |  |  |  |  |  |  |  |
| e2 (additive) | -0.029 | 0.020 | 0.151 | -0.036 | 0.020 | 0.075 | 0.019 | 0.018 | 0.277 | -0.005 | 0.019 | 0.786 |
| e2 (yes vs no) | -0.032 | 0.021 | 0.134 | -0.039 | 0.022 | 0.070 | 0.025 | 0.019 | 0.182 | -0.004 | 0.020 | 0.861 |
|  |  |  |  |  |  |  |  |  |  |  |  |  |
| Age >60 yrs |  |  |  |  |  |  |  |  |  |  |  |  |
| e2 (additive) | -0.037 | 0.043 | 0.390 | -0.060 | 0.044 | 0.173 | 0.020 | 0.040 | 0.615 | 0.024 | 0.044 | 0.580 |
| e2 (yes vs no) | -0.046 | 0.046 | 0.314 | -0.057 | 0.046 | 0.219 | 0.030 | 0.042 | 0.472 | 0.026 | 0.046 | 0.573 |
|  |  |  |  |  |  |  |  |  |  |  |  |  |
| Age ≤60 yrs |  |  |  |  |  |  |  |  |  |  |  |  |
| e2 (additive) | -0.021 | 0.022 | 0.350 | -0.025 | 0.023 | 0.264 | 0.022 | 0.021 | 0.291 | -0.012 | 0.021 | 0.571 |
| e2 (yes vs no) | -0.022 | 0.024 | 0.345 | -0.027 | 0.024 | 0.264 | 0.028 | 0.022 | 0.209 | -0.010 | 0.022 | 0.648 |

Beta: Standardised beta, SE: Standard Error, P: Incremental P-value.

Supplementary Table 4: Associations between *APOE* and general cognitive ability adjusted for age and sex.

|  |  | g |  | g (age > 60) | | | g (age <= 60) | | |
| --- | --- | --- | --- | --- | --- | --- | --- | --- | --- |
|  | Beta | SE | P | Beta | SE | P | Beta | SE | P |
| e4 (additive) | 0.022 | 0.015 | 0.137 | -0.040 | 0.032 | 0.221 | 0.028 | 0.017 | 0.094 |
|  |  |  |  |  |  |  |  |  |  |
| e4 (yes vs no) | 0.032 | 0.017 | 0.063 | -0.038 | 0.037 | 0.306 | **0.039** | **0.019** | **0.039** |
|  |  |  |  |  |  |  |  |  |  |
| e2e2 | -0.061 | 0.093 | 0.500 | -0.111 | 0.220 | 0.664 | -0.067 | 0.104 | 0.461 |
| e2e3 | -0.010 | 0.024 | 0.436 | -0.029 | 0.050 | 0.741 | 0.003 | 0.027 | 0.778 |
| e2e4 | -0.016 | 0.049 | 0.559 | -0.040 | 0.111 | 0.705 | -0.011 | 0.054 | 0.608 |
| e3e4 | **0.037** | **0.018** | **0.038** | -0.035 | 0.040 | 0.461 | **0.047** | **0.020** | **0.017** |
| e3e3 | - | - | - | - | - | - | - | - | - |
| e4e4 | -0.007 | 0.046 | 0.884 | -0.127 | 0.107 | 0.237 | -0.002 | 0.051 | 0.972 |

g: general cognitive ability, Beta: Standardised beta, SE: Standard Error, P: Incremental P-value. Supplementary Table 5: Interaction association between *APOE*:age and cognitive ability adjusted for sex.

|  | **Logical Memory** | | | **Verbal Fluency Test** | | | **Digit Symbol Test** | | | **Mill Hill Vocabulary Scale** | | |
| --- | --- | --- | --- | --- | --- | --- | --- | --- | --- | --- | --- | --- |
|  | Beta | SE | P | Beta | SE | P | Beta | SE | P | Beta | SE | P |
| e4 (additive) | -0.010 | 0.014 | 0.480 | **0.037** | **0.014** | **0.008** | 0.012 | 0.012 | 0.339 | 0.010 | 0.013 | 0.419 |
|  |  |  |  |  |  |  |  |  |  |  |  |  |
| e4 (yes vs no) | -0.007 | 0.016 | 0.673 | **0.033** | **0.016** | **0.034** | 0.018 | 0.014 | 0.196 | 0.011 | 0.014 | 0.436 |

Beta: Standardised beta, SE: Standard Error, P: Incremental P-value.

Supplementary Table 6: Associations between *APOE* and cognitive ability adjusted for age, sex, education, social class, and self-reported heart disease, history of stroke, high blood pressure, diabetes, Alzheimer's disease, and depression.

|  | **Logical Memory** | | | **Verbal Fluency Test** | | | **Digit Symbol Test** | | | **Mill Hill Vocabulary Scale** | | |
| --- | --- | --- | --- | --- | --- | --- | --- | --- | --- | --- | --- | --- |
|  | Beta | SE | P | Beta | SE | P | Beta | SE | P | Beta | SE | P |
| e4 (additive) | **-0.031** | **0.015** | **0.033** | **0.057** | **0.015** | **<0.001** | -0.015 | 0.013 | 0.230 | **0.028** | **0.013** | **0.028** |
|  |  |  |  |  |  |  |  |  |  |  |  |  |
| e4 (yes vs no) | -0.023 | 0.017 | 0.167 | **0.061** | **0.017** | **<0.001** | -0.015 | 0.014 | 0.302 | **0.033** | **0.015** | **0.024** |
|  |  |  |  |  |  |  |  |  |  |  |  |  |
| e2e2 | -0.006 | 0.094 | 0.912 | -0.016 | 0.094 | 0.808 | -0.019 | 0.081 | 0.755 | -0.014 | 0.082 | 0.765 |
| e2e3 | -0.033 | 0.023 | 0.304 | -0.018 | 0.024 | 0.127 | 0.031 | 0.020 | 0.077 | 0.007 | 0.021 | 0.851 |
| e2e4 | -0.046 | 0.049 | 0.477 | -0.039 | 0.049 | 0.178 | -0.013 | 0.042 | 0.822 | 0.038 | 0.043 | 0.556 |
| e3e4 | -0.016 | 0.018 | 0.675 | **0.063** | **0.018** | **0.002** | -0.007 | 0.015 | 0.762 | 0.033 | 0.016 | 0.052 |
| e3e3 | - | - | - | - | - | - | - | - | - | - | - | - |
| e4e4 | **-0.141** | **0.046** | **0.002** | **0.109** | **0.046** | **0.019** | -0.036 | 0.040 | 0.368 | 0.041 | 0.041 | 0.313 |

Beta: Standardised beta, SE: Standard Error, P: Incremental P-value.

Supplementary Table 7. Age-stratified (>60 and ≤60) associations between *APOE* and cognitive ability adjusted for age, sex, education, social class, and self-reported heart disease, history of stroke, high blood pressure, diabetes, Alzheimer's disease, and depression.

| Age >60 yrs | **Logical Memory** | | | **Verbal Fluency Test** | | | **Digit Symbol Test** | | | **Mill Hill Vocabulary Scale** | | |
| --- | --- | --- | --- | --- | --- | --- | --- | --- | --- | --- | --- | --- |
|  | Beta | SE | P | Beta | SE | P | Beta | SE | P | Beta | SE | P |
| e4 (additive) | **-0.083** | **0.033** | **0.011** | **0.088** | **0.032** | **0.007** | **-0.073** | **0.029** | **0.013** | -0.007 | 0.029 | 0.815 |
|  |  |  |  |  |  |  |  |  |  |  |  |  |
| e4 (yes vs no) | **-0.075** | **0.037** | **0.044** | **0.083** | **0.037** | **0.025** | **-0.066** | **0.033** | **0.046** | -0.006 | 0.034 | 0.859 |
|  |  |  |  |  |  |  |  |  |  |  |  |  |
| e2e2 | 0.054 | 0.227 | 0.694 | -0.231 | 0.225 | 0.286 | -0.215 | 0.202 | 0.321 | 0.024 | 0.204 | 0.921 |
| e2e3 | -0.075 | 0.051 | 0.326 | -0.065 | 0.050 | 0.080 | 0.017 | 0.045 | 0.413 | 0.024 | 0.046 | 0.580 |
| e2e4 | -0.119 | 0.112 | 0.392 | -0.041 | 0.111 | 0.856 | -0.089 | 0.099 | 0.475 | -0.052 | 0.101 | 0.600 |
| e3e4 | -0.061 | 0.041 | 0.224 | 0.053 | 0.041 | 0.299 | -0.043 | 0.036 | 0.359 | 0.005 | 0.037 | 0.863 |
| e3e3 | - | - | - | - | - | - | - | - | - | - | - | - |
| e4e4 | **-0.298** | **0.108** | **0.006** | **0.264** | **0.107** | **0.014** | **-0.246** | **0.096** | **0.011** | -0.022 | 0.098 | 0.825 |
|  |  |  |  |  |  |  |  |  |  |  |  |  |
| Age ≤60 yrs | **Logical Memory** | | | **Verbal Fluency Test** | | | **Digit Symbol Test** | | | **Mill Hill Vocabulary Scale** | | |
|  | Beta | SE | P | Beta | SE | P | Beta | SE | P | Beta | SE | P |
| e4 (additive) | -0.020 | 0.016 | 0.221 | **0.044** | **0.016** | **0.006** | -0.010 | 0.015 | 0.522 | **0.031** | **0.014** | **0.030** |
|  |  |  |  |  |  |  |  |  |  |  |  |  |
| e4 (yes vs no) | -0.012 | 0.018 | 0.511 | **0.052** | **0.018** | **0.005** | -0.010 | 0.017 | 0.539 | **0.035** | **0.016** | **0.029** |
|  |  |  |  |  |  |  |  |  |  |  |  |  |
| e2e2 | -0.005 | 0.104 | 0.978 | -0.013 | 0.104 | 0.819 | 0.004 | 0.096 | 0.962 | -0.039 | 0.091 | 0.590 |
| e2e3 | -0.018 | 0.026 | 0.613 | 3.7x10^-5^ | 0.026 | 0.564 | 0.042 | 0.024 | 0.062 | 1.3x10^-4^ | 0.023 | 0.602 |
| e2e4 | -0.025 | 0.055 | 0.726 | -0.044 | 0.054 | 0.217 | -0.013 | 0.050 | 0.811 | 0.058 | 0.048 | 0.338 |
| e3e4 | -0.005 | 0.020 | 0.952 | **0.060** | **0.020** | **0.004** | -0.002 | 0.018 | 0.960 | 0.032 | 0.018 | 0.098 |
| e3e3 | - | - | - | - | - | - | - | - | - | - | - | - |
| e4e4 | **-0.107** | **0.051** | **0.037** | 0.060 | 0.051 | 0.240 | -0.011 | 0.047 | 0.810 | 0.048 | 0.045 | 0.287 |

Beta: Standardised beta, SE: Standard Error, P: Incremental P-value.
